
JOURNAL INFORMATION
==============================
NLM Title Abbreviation: Tex Med J (Austin)
NLM Journal ID: 21130590R

Texas Medical Journal

ISSN: 0892-8495
Publisher: Biomedical Journal Digitization Project

ARTICLE INFORMATION
==============================
PMCID: PMC9590971
Article version: 1
Subjects: Books and Magazines

Atlas and Essentials of Pathological Anatomy

Print publication date: 1899 Feb
Volume: 14
Issue: 8
First page: 472
Last page: 472
License: This work is free of known copyright restrictions. For more information, please see PMC Back Issue Digitization.
License URL: https://creativecommons.org/publicdomain/mark/1.0/

==============================

ATLAS AND ESSENTIALS OF PATHOLOGICAL ANATOMY.—By Dr. O. Ballinger, Obermedicinalrat and Professor. Vol. II. Urinary apparatus, sexual organs, nervous system and bones, with 63 colored figures upon 52 plates and 17 illustrations in the text. Published by Messrs. Wm. Wood & Co., New York. 1898. Price, cloth, $3.00 net.

This, the second volume of this valuable work, is in every way equal to the first volume elsewhere reviewed. The two together form a very valuable, complete and beautiful work on pathological anatomy, and should be in every physician's library. I. J. J.

---

### Publisher's Notes.

---

TUSCUMBIA, ALA., July 2, 1898.

*The Vass Chemical Co., Danbury, Conn.*

GENTLEMENS—I have used one bottle of Thialion with *great satisfaction* in a case of gravel of long standing. You will please find P. O. money order for $1.00 for which send me another bottle and oblige. Very respectfully yours,

A. ALVA SENSABAUGH, M. D.

---

I have prescribed Seng successfully on several occasions, and I regard it as a valuable remedy when used in suitable cases. In one obstinate case in particular, it has proved of greater service than anything formerly prescribed, and I shall revert to it in future with increased confidence in the result.

H. H. BIRKETT WILKINSON, M. D.,
L. R. C. P., L. R. C. S., L. M.

South Grove, Rotherham, Eng.

---

FIKULAX is delightful in taste and attractive in appearance, important attributes in a medicament to be administered to women and children.

Fikulux contains Pimenta, oleum cassiæ, ext. cascaræ sag., magnesia gravis, sulphuris, rhamnus frangula, ext. glycyrrhizæ, ext. sennæ fluidum, zingiberis, cinnamomum, cardamomum, myristica, ficus celestia et chocolata.

Fikulax is put up in boxes containing 12.

---

SANMETTO A STANDARD REMEDY IN GENITO-URINARY DISEASES.—I have prescribed Sanmetto in a large number of cases of genito-urinary troubles during the past four years, and with uniformly good success. In prostatic troubles of old men, with difficult micturition, it acts like a charm. In cases of irritable bladder with incontinence of urine, I have never met with any remedy that acts so
